# Genetic Deletion of Polo-Like Kinase 2 Induces a Pro-Fibrotic Pulmonary Phenotype

**DOI:** 10.3390/cells10030617

**Published:** 2021-03-11

**Authors:** Theresa A. Kant, Manja Newe, Luise Winter, Maximilian Hoffmann, Susanne Kämmerer, Erik Klapproth, Karolina Künzel, Mark P. Kühnel, Lavinia Neubert, Ali El-Armouche, Stephan R. Künzel

**Affiliations:** 1 Institute of Pharmacology and Toxicology, Faculty of Medicine Carl Gustav Carus, Technische Universität Dresden, 01307 Dresden, Germany; theresa.kant@tu-dresden.de (T.A.K.); manja.newe@tu-dresden.de (M.N.); luisewinter@mailbox.org (L.W.); maximilian.hoffmann@tu-dresden.de (M.H.); susanne.kaemmerer@tu-dresden.de (S.K.); erik.klapproth@tu-dresden.de (E.K.); karolina.kuenzel@mailbox.tu-dresden.de (K.K.); 2 Institute of Pathology, Hannover Medical School, 30625 Hannover, Germany; kuehnel.mark@mh-hannover.de (M.P.K.); neubert.lavinia@mh-hannover.de (L.N.)

**Keywords:** pulmonary fibrosis, PLK2, fibroblasts

## Abstract

Pulmonary fibrosis is the chronic-progressive replacement of healthy lung tissue by extracellular matrix, leading to the destruction of the alveolar architecture and ultimately death. Due to limited pathophysiological knowledge, causal therapies are still missing and consequently the prognosis is poor. Thus, there is an urgent clinical need for models to derive effective therapies. Polo-like kinase 2 (PLK2) is an emerging regulator of fibroblast function and fibrosis. We found a significant downregulation of *PLK2* in four different entities of human pulmonary fibrosis. Therefore, we characterized the pulmonary phenotype of PLK2 knockout (KO) mice. Isolated pulmonary PLK2 KO fibroblasts displayed a pronounced myofibroblast phenotype reflected by increased expression of αSMA, reduced proliferation rates and enhanced ERK1/2 and SMAD2/3 phosphorylation. In PLK2 KO, the expression of the fibrotic cytokines *osteopontin* and *IL18* was elevated compared to controls. Histological analysis of PLK2 KO lungs revealed early stage remodeling in terms of alveolar wall thickening, increased alveolar collagen deposition and myofibroblast foci. Our results prompt further investigation of PLK2 function in pulmonary fibrosis and suggest that the PLK2 KO model displays a genetic predisposition towards pulmonary fibrosis, which could be leveraged in future research on this topic.

## 1. Introduction

Pulmonary fibrosis encompasses multiple pathological conditions of the lungs in which functional alveolar tissue is progressively replaced by extracellular matrix [1,2]. The nature of pulmonary fibrosis is highly heterogeneous with respect to disease-driving stimuli (e.g., smoking, radiation, dusts or drugs), pathological features and clinical outcome [1,2,3,4]. Nonetheless, there are common motifs among the different entities of pulmonary fibrosis such as alveolar wall thickening due to collagen deposition, inflammation and fibroblast activation [1,2,5]. Patients suffer from impaired gas exchange, increased pulmonary resistance [1] and impaired cardiac function [6,7]. To date therapeutic options to stop or reverse fibrosis are limited due to an incomplete understanding of the underlying pathophysiology [8].

Animal models have been developed to investigate cellular and molecular pulmonary fibrosis mechanisms. Common animal models of pulmonary fibrosis include the application of chemical irritants like bleomycin or FITC, irradiation, the instillation of silica dust, transgenic mouse lines or adoptive cell transfer [3]. Although the use of animals has increased the understanding of pulmonary fibrosis, there is currently no model, which recapitulates all characteristic pathological features of the human disease [3]. Therefore, the introduction of new models recapitulating distinct pathophysiological hallmarks can make valuable contributions to a broader understanding of human pulmonary fibrosis and thus support the development of new therapies.

Polo-like kinase 2 (PLK2) is a serine-threonine kinase that regulates cell cycle progression, cell proliferation, mitochondrial respiration and apoptosis [9,10,11,12]. There is first evidence that PLK2 regulates fibrosis formation in cardiovascular disease [13] and differential regulation of *PLK2* gene expression was found in Transforming Growth Factor beta 1 (TGF-β) treated adult lung fibroblasts [14]. However, the function of PLK2 in pulmonary fibrosis development remains unclear.

The objective of the present study was to examine *PLK2* gene expression in different entities of human pulmonary fibrosis and to characterize the pulmonary phenotype of *PLK2* deficient mice. We provide experimental evidence linking *PLK2* knockout (KO) to fibroblast dysfunction, increased gene expression of the pro-fibrotic cytokines *osteopontin* (OPN) and *interleukin 18* (IL18) [15,16,17] and early stage histological remodeling.

## 2. Materials and Methods

### 2.1. PLK2 Mice

PLK2 wild type (WT) and KO mice (129S.B6N-*Plk2^tm1Elan^*/J, stock number: 017001 Plk2 KO) are commercially available via The Jackson Laboratory (The Jackson Laboratory, Bar Harbor, ME USA). PLK2 WT and KO mice were generated from heterozygous (PLK2^+/–^) breeding couples to ensure that both groups display the same genetic background. To study the general effects of PLK2 deficiency and to reduce animal numbers, male and female mice at 7–8 months of age were included in this study.

### 2.2. Human Sample Acquisition

Anonymized human lung specimens (Table 1) were obtained from the biobank of the project BREATH by the Institute of Pathology at Medizinische Hochschule Hannover, Germany. Native lung tissue was obtained from whole lung explanation and immediately processed within 20–30 min following standard operating procedures [18,19]. Tissue from both lungs was used for clinical diagnostic purposes. Additionally, left lung tissue was histologically processed and stored in the biobank for research.

### 2.3. SDS-PAGE, Western Blotting and Immunodetection

For protein extraction, 12.5 × 10^4^ cells per well were seeded onto 6-well plates and harvested after 72 h. Therefore, cells were washed once with cold PBS, and 56 µL of radioimmunoprecipitation assay buffer (30 mM Tris, 0.5 mM EDTA, 150 mM NaCl, 1% NP-40, 0.1% SDS) supplemented with 10% protease and phosphatase inhibitors (Hoffmann-La Roche, Basel, Switzerland) was added directly to each well and cells were scraped off. Protein concentration was determined with a bicinchoninic acid kit (Thermo Fisher Scientific, Waltham, MA USA). Western blots were performed as described previously [8,20]. 10 µg of whole cell protein was separated on a 10% polyacrylamide gel and subsequently transferred to a nitrocellulose membrane. Immuno-detection was performed with a Fusion FX device (Vilber Lourmat Deutschland GmbH, Eberhardzell, Germany). Table 2 summarizes all antibodies and respective concentrations used in this study.

### 2.4. RNA Isolation, cDNA Synthesis and qPCR

SYBR green (Bio-Rad Laboratories GmbH, Munich, Germany) real-time PCR was performed to measure the gene expression of *PLK2, OPN* and *IL18*. Specific primers were purchased from Bio-Rad (Bio-Rad Laboratories GmbH, Munich, Germany). *GAPDH* was used as housekeeping gene. For RNA isolation from murine lung tissue the RNeasy mini kit (QIAGEN GmbH, Hilden, Germany) was used according to the manufacturer’s instructions. For RNA isolation from paraffin embedded human lung tissue samples the miRNeasy FFPE kit (QIAGEN GmbH, Hilden, Germany) was used according to the manufacturer’s instructions. Per human sample, two 10 µm sections of paraffin embedded tissue were processed for RNA isolation. Subsequent cDNA synthesis was performed with the PeqGold cDNA synthesis kit (Peqlab Biotechnologie GmbH, Erlangen, Germany). qPCR runs were performed in a CFX96 Touch Deep Well Real-Time PCR detection system (Bio-Rad Laboratories GmbH, Munich, Germany). Samples were amplified in duplicates. For data analysis the CFX manager software (Bio-Rad Laboratories GmbH, Munich, Germany) was used. Relative gene expression was calculated to housekeeping gene and normalized to control.

### 2.5. Primary Fibroblast Isolation

The isolation of primary PLK2 WT and KO fibroblasts was performed as described recently[21].

### 2.6. Human Pulmonary MRC5 Fibroblasts

The human pulmonary fibroblast cell line MRC5 (ATCC^®^ CCL-171™) was purchased from ATCC (American Type Culture Collection (ATCC), Manassas, VA USA).

### 2.7. Cell Culture

Primary fibroblasts were cultured at 37 °C and 5% CO_2_ in Dulbecco’s modified eagle medium (Life Technologies, Carlsbad, CA, USA) supplemented with 10 % fetal calf serum (FCS, Life Technologies, Carlsbad, CA, USA) and 1 % penicillin/ streptomycin (Life Technologies, Carlsbad, CA, USA). The cell culture medium was changed every other day.

### 2.8. Fibroblast Immunofluorescence Staining

For primary fibroblasts, 1 × 10^4^ cells/ well were seeded on glass coverslips in 24-well plates. Cells were cultured until optical confluence of 80–90% was achieved (4 ± 1 days). Subsequently, cells were washed twice with PBS and fixed with 4% paraformaldehyde (15 min at room temperature). The following steps were performed after fixation (Table 3).

### 2.9. Extracellular Collagen Deposition

For assessment of PLK2-dependent collagen deposition, 1 × 10^4^ cells/cm^2^ were seeded on borosilicate glass coverslips in standard culture medium (see above). After 24 h, the medium was changed to DMEM supplemented with 0.5% FCS, 1% penicillin/streptomycin, 0.5 mmol/L ascorbic acid and PLK2 inhibitor (TC-S 7005, Tocris Bioscience) or solvent control (1 µL/ mL DMSO). After 48 h, cells were fixed in 4% para-formaldehyde. Collagen deposition was visualized with ICC against collagen Iα1. For image analysis, FIJI [22] was used. The overall collagen-covered area was normalized to the number of nuclei in the respective image.

### 2.10. Apoptosis Detection

For apoptosis detection, the Click-iT™ Plus TUNEL Assay was purchased and used according to the manufacturer’s instructions. For quantification, CellProfiler V4.1.3 [23] was used. Green fluorescence intensity was normalized to the number of nuclei per image.

### 2.11. Fibroblast Proliferation

1 × 10^4^ cells/ well were seeded in 12-well plates. The medium was changed every other day. Cells were harvested and counted after 7 and 14 days using 0.25% trypsin and a Buerker counting chamber with trypan blue dye. Results were calculated as cells × 10^4^/mL.

### 2.12. Histology and Imaging

For histological analysis, mouse lung tissue was fixed in 4% paraformaldehyde overnight. Subsequent paraffin embedding, sectioning (5 µm) and staining (hematoxylin/eosin and picrosirius red) was performed by the histology facility at the Center for Molecular and Cellular Bioengineering (CMCB) Dresden. For immunohistochemical staining deparaffinization in serial solutions of xylene, ethanol and water were followed by antigen retrieval via steaming in 10 mM citric acid (pH 6.0) for 20 min and a subsequent cooling period of 20 min at room temperature. Samples were washed twice with PBS for 10 min and subsequently blocked with 10% FCS for 60 min. Following, samples were incubated with anti-α-SMA antibody overnight at 4 °C in a humidified chamber. After washing twice with PBS, the samples were incubated with the secondary antibody (Alexa fluor 546; Thermo Fisher Scientific, Waltham, MA USA) and DAPI for 60 min. Fluorescence and brightfield images (for picrosirius red) of randomly chosen areas were acquired with a Keyence BZ-X710 All-in-One Fluorescence Microscope (Keyence Corporation of America, Itasca, IL USA). Fibrosis and fluorescence intensity were quantified using FIJI 1.52n software (Wayne Rasband, National Institutes of Health, Bethesda, MD USA).

### 2.13. Units

SI units were used throughout the manuscript whenever possible. In the case of a unitless result, the measure “arbitrary units” (AU) was applied.

### 2.14. Statistical Analysis

All results are presented as mean ± SEM. For statistical analysis and graphic representation of the data, Graph Pad Prism software v.8 (GraphPad Software, San Diego, CA USA) was used. Comparisons between two groups were made using Student’s t-test with Welsh’s correction if appropriate. For comparisons of three or more groups, one-way ANOVA with Tukey posttest was performed. *p* < 0.05 was considered statistically significant (* *p* < 0.05; ** *p* < 0.01; *** *p* < 0.001).

## 3. Results and Discussion

### 3.1. PLK2 Expression Is Reduced in Human Pulmonary Fibrosis

Fibrosis development is a complex pathological process with tremendous variability depending on the site of manifestation [2]. Pulmonary fibrosis comprises a particularly heterogeneous group of fibroproliferative disease, which can be the sequelae of multiple triggering stimuli [1,24]. Recent findings suggest that PLK2 is involved in non-pulmonary fibrosis regulation[13] and differential PLK2 expression has been demonstrated in pulmonary fibroblasts upon stimulation with TGFβ [14]. To ascertain whether PLK2 and its downstream targets are differentially regulated in pulmonary fibrosis, we analyzed gene expression in lung tissue samples from different fibrotic entities such as idiopathic pulmonary fibrosis (IPF), alveolar fibroelastosis (AFE), organizing pneumonia (OP) and systemic sclerosis (SSC). We found significantly lower *PLK2* expression in all fibrotic specimen compared to control (Table 4). Although the sample size is relatively small due to the limited availability of human lung specimen, these results indicate that reduced *PLK2* gene expression could be a common motif of human pulmonary fibrosis. Interestingly, there is growing consent that generally valid, unifying fibrosis mechanisms and common pathogenic pathways exist [2,25]. Thus, identification of targets relevant in multiple entities of fibrosis could open up pathways towards universally applicable antifibrotic treatment.

### 3.2. Primary PLK2 KO Fibroblasts Display a Myofibroblast Phenotype

Fibroblasts are considered the major cellular regulators of fibrosis [26,27]. Under physiological conditions, fibroblasts maintain the homeostasis of the extracellular matrix (ECM) by secretion and degradation of collagens and ECM proteins [26]. Upon exposure towards activating stimuli like mechanical injury, cytokine exposure or irradiation [2,26], fibroblasts undergo a phenotype conversion to myofibroblasts, which are characterized by the expression of contractile α-smooth muscle actin (αSMA) fibers [8].

**Figure 1:**
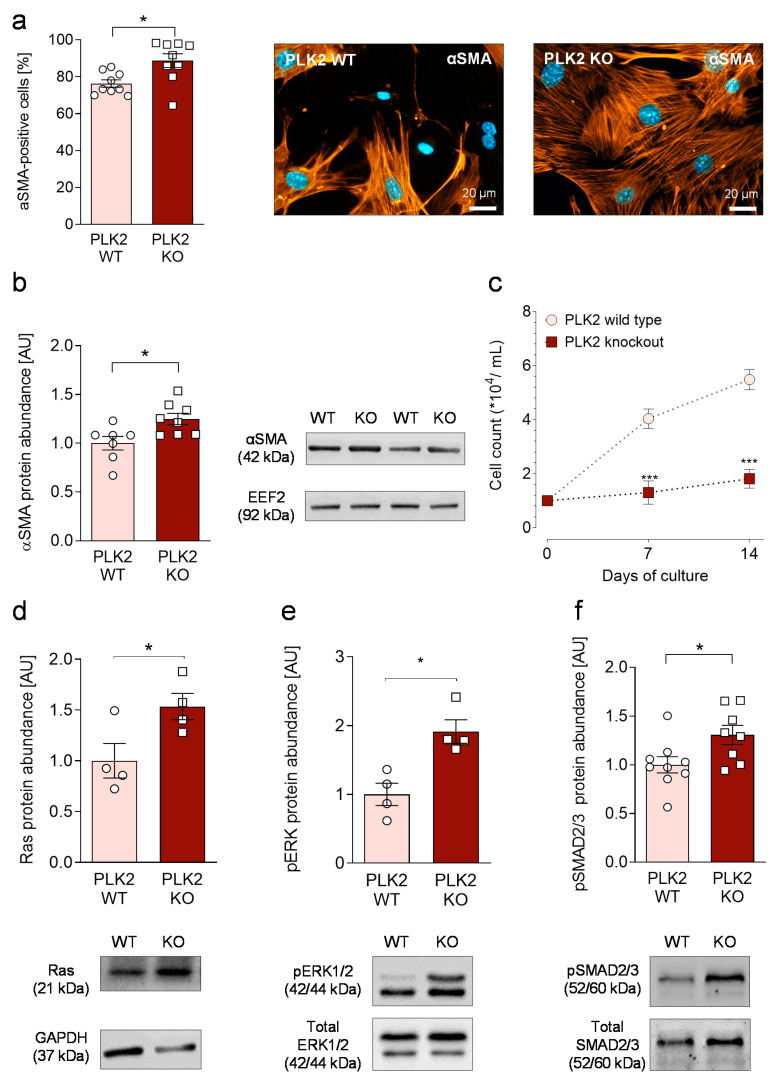
Characterization of the pulmonary fibroblast phenotype in PLK2 WT and KO. (**a**) Quantification and representative immunofluorescence images of αSMA myofilaments in primary PLK2 WT and KO fibroblasts (*n_WT_* = 9, *n_KO_* = 9). The nuclei were stained with DAPI (blue). The scale bars equal 20 µm. (**b**) Quantification and representative Western blot for αSMA in primary PLK2 WT and KO fibroblasts (*n_WT_* = 7, *n_KO_* = 8). (**c**) Proliferation curves of primary pulmonary PLK2 WT and KO fibroblasts under control conditions (*n_WT_* = 14 from 7 animals, *n_KO_* = 10 from 5 animals). Cells were counted after 7 and 14 days. (**d**) Quantification and representative Western blot for Ras (*n_WT_* = 4, *n_KO_* = 4). (**e**) Quantification and representative Western blot for ERK1/2 phosphorylation (*n_WT_* = 4, *n_KO_* = 4). (**f**) Quantification and representative Western blot for SMAD2/3 phosphorylation (*n_WT_* = 9, *n_KO_* = 8). * *p* < 0.05. *** *p* < 0.001.

To investigate whether reduced or absent PLK2-function contributes to fibrosis development; primary pulmonary fibroblasts were isolated from PLK2 WT and KO mice. All isolated cells expressed accepted fibroblast marker proteins such as Vimentin, Collagen 1 and DDR2 [28] (Figure S1). Myofibroblast differentiation was determined by the expression of αSMA filaments. Cells were grown on glass coverslips and a minimum of 50 cells per coverslip was analyzed. Although pronounced spontaneous myofibroblast differentiation was present in WT, the expression of αSMA filaments was significantly elevated in PLK2 KO fibroblasts (Figure 1a). Fibroblasts represent a heterogeneous cell population and differences in the extent of spontaneous myofibroblast differentiation in vitro have been reported with high levels in pulmonary fibroblasts [29]. Moreover, spontaneous differentiation can be a cell culture artifact due to, e.g., non-physiological surface rigidity of culture dishes [30,31]. Previous work of our group focused on primary human cardiac fibroblasts in sinus rhythm and atrial fibrillation. Although high spontaneous differentiation rates were encountered in the control group, discrete differences of approximately 20% were preserved in atrial fibrillation and considered relevant [32]. In the present study, elevated differentiation in PLK2 KO was confirmed by an increase in αSMA protein abundance determined by Western blot (Figure 1b, Figure S3). Cell proliferation on the other side was significantly reduced in PLK2 KO (Figure 1c), suggesting an inverse correlation of fibroblast differentiation and proliferation, which has also been reported in cardiac myofibroblasts [27,32]. Mechanistically, the phosphorylation of SMAD2/3 is considered a crucial step in fibroblast activation and fibrosis formation [33,34]. Hence, we studied mediators of SMAD2/3 phosphorylation in PLK2 WT and KO fibroblasts. We found increased protein expression of Ras and enhanced ERK1/2 phosphorylation (Figure 1d,e)—Both mediators are reported to act upstream of SMAD2/3 [35,36,37,38]. Accordingly, SMAD2/3 phosphorylation was significantly elevated in KO compared to WT (Figure 1f). These results are in line with our previous finding, that genetic deletion and pharmacological inhibition of PLK2 stimulate ERK1/2 phosphorylation in cardiac fibroblasts [13]. Although the mechanisms by which PLK2 modulates these pathways are little understood, there is data demonstrating that neuronal PLK2 regulates the Ras pathway by phosphorylation-dependent degradation of RasGRF1, a guanidine exchange factor that stimulates Ras activity [39,40]. Taken together our results indicate spontaneous myofibroblast differentiation in PLK2 KO fibroblasts potentially mediated by activation of the Ras-ERK1/2-SMAD2/3 pathway. However, in order to draw definitive mechanistic conclusions, the interactions of PLK2 with Ras, ERK1/2 and SMAD2/3 need to be specifically investigated in future projects.

### 3.3. Expression of OPN and IL18 Is Elevated in PLK2 KO and Human IPF and AFE

**Figure 2:**
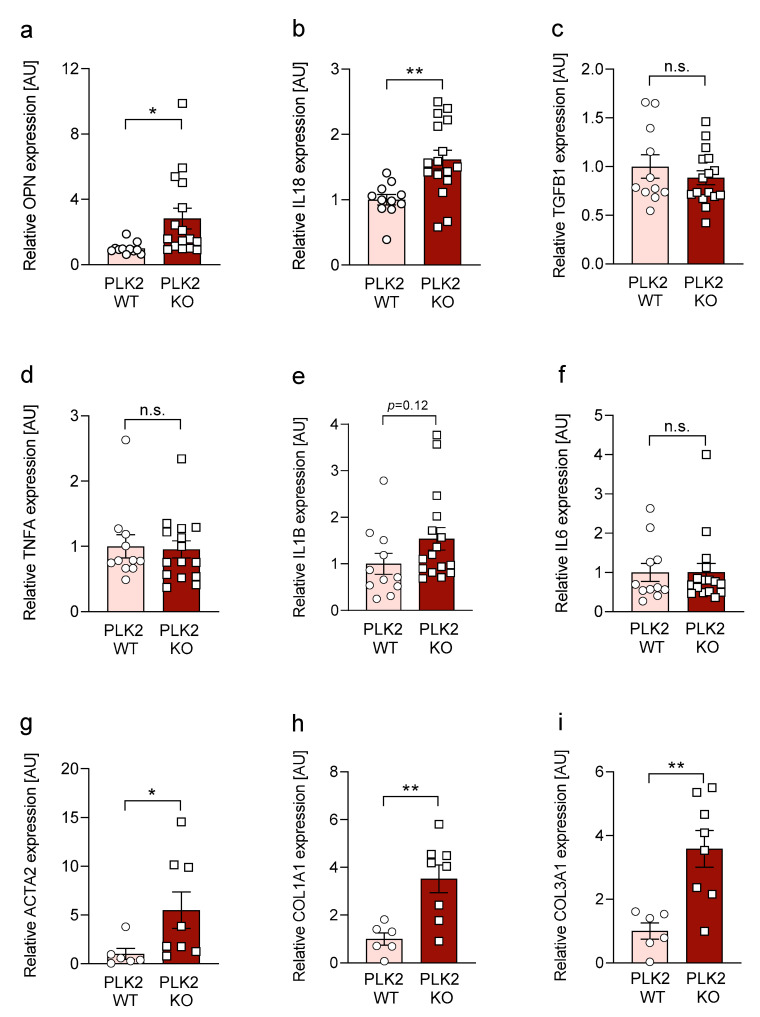
Analysis of pro-fibrotic gene expression with RT qPCR in PLK2 WT and KO lung specimens. (**a**) Quantification of *OPN* gene expression in PLK2 WT and KO lung tissue normalized to WT (*n_WT_* = 11, *n_KO_* = 16). (**b**) Quantification of *IL18* gene expression in PLK2 WT and KO lung tissue normalized to WT (*n_WT_* = 11, *n_KO_* = 16). (**c**) Quantification of *TGFB1* gene expression in PLK2 WT and KO lung tissue normalized to WT (*n_WT_* = 11, *n_KO_* = 16). (**d**) Quantification of *TNFA* gene expression in PLK2 WT and KO lung tissue normalized to WT (*n_WT_* = 11, *n_KO_* = 16). (**e**) Quantification of *IL1B* gene expression in PLK2 WT and KO lung tissue normalized to WT (*n_WT_* = 11, *n_KO_* = 16). (**f**) Quantification of *IL6* gene expression in PLK2 WT and KO lung tissue normalized to WT (*n_WT_* = 11, *n_KO_* = 16). (**g**) Quantification of *ACTA2 (αSMA)* gene expression in PLK2 WT and KO lung tissue normalized to WT (*n_WT_* = 6, *n_KO_* = 8). (**h**) Quantification of *COL1A1* gene expression in PLK2 WT and KO lung tissue normalized to WT (*n_WT_* = 6, *n_KO_* = 8). (**i**) Quantification of *COL3A1* gene expression in PLK2 WT and KO lung tissue normalized to WT (*n_WT_* = 6, *n_KO_* = 8). * *p* < 0.05. ** *p* < 0.01. n.s. = not significant.

The expression of pro-fibrotic cytokines is closely intertwined with inflammation and fibrosis development [2]. Among others Transforming Growth Factor beta 1 (TGF-β) [41], OPN [15,16], Tumor Necrosis Factor alpha (TNFα) [42], and members of the interleukin family (i.e., IL1β, IL6 and IL18) [17,43,44] are known drivers of (pulmonary) fibrosis. In PLK2 KO lung tissue, we found a significant upregulation of *OPN* and *IL18* gene expression (Figure 2a,b) while *TGF-β, TNFα, IL1β* and *IL6* were not differentially upregulated. In accordance with the myofibroblast phenotype present in PLK2 KO (Figure 1), the expression of *ACTA2 (*αSMA), *Col1A1* and *Col3A1* was significantly upregulated compared to WT (Figure 2g,h,i). Based on these findings, we focused on *OPN* and *IL18* for further investigation in human pulmonary fibrosis samples. IPF and AFE patient samples showed a clear trend of *OPN* and *IL18* upregulation (Figure S2a,b). However, the results did not reach the level of statistical significance. Nonetheless, the observed trend is in agreement with published data, demonstrating a disease-driving role of OPN in IPF [17,45,46]. Additionally, these results support previous findings that both genetic deletion and pharmacological inhibition of PLK2 lead to a significant increase in OPN protein expression in cardiac fibroblasts [13]. However, as of now, it remains the subject of future research whether fibroblasts, alveolar epithelial cells, immune cells or their specific interactions are accountable for the observed increase of *OPN* and *IL18* expression in PLK2 KO lungs.

### 3.4. Histological Analysis of PLK2 KO Lungs

**Figure 3:**
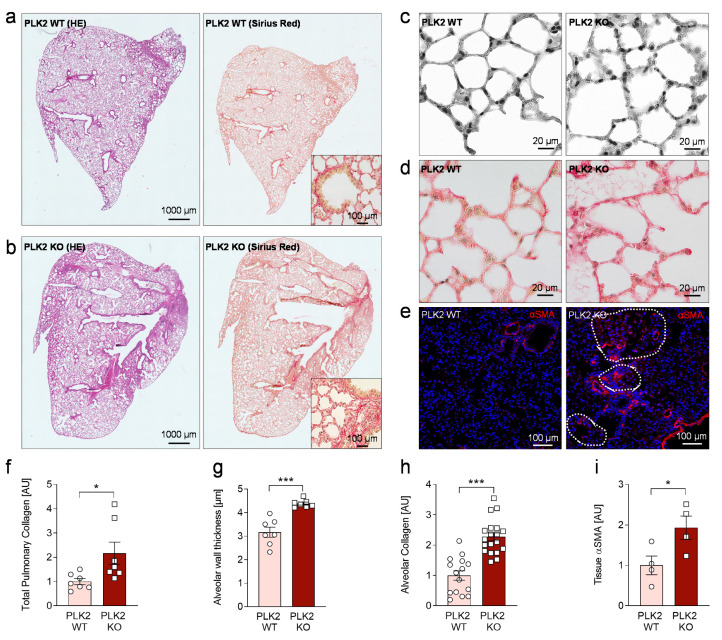
Histological analysis of PLK2 WT and KO lung sections. (**a**,**b**) Representative images of hematoxylin/ eosin and picrosirius red collagen staining in PLK2 WT and KO lung sections. The scale bars equal 1000 and 100 µm for the inlets, respectively. (**c**) Representative images of PLK2 WT and KO alveolar tissue (black and white for better contrast visualization). The scale bars equal 20 µm. (**d**) Representative picrosirius red images of PLK2 WT and KO alveolar collagen. The scale bars equal 20 µm. (**e**) Representative immunofluorescence images of αSMA (red) and nuclei (blue) in PLK2 WT and KO lung sections. Highlighted areas (dashed white outline) show myofibroblast accumulation (foci). The scale bars equal 100 µm. (**f**) Quantification of total pulmonary collagen content in PLK2 WT and KO lung sections (*n_WT_* = 7, *n_KO_* = 7). (**g**) Quantification of alveolar wall thickness [µm] (*n_WT_* = 7, *n_KO_* = 7). (**h**) Quantification of alveolar collagen in PLK2 WT and KO lung sections (*n_WT_* = 15 analyzed imaged from 5 animals, *n_KO_* = 19 analyzed images from 6 animals). (**i**) Quantification of αSMA fluorescence intensity (normalized to nuclei) in PLK2 WT and KO lung sections (*n_WT_* = 4, *n_KO_* = 4). * *p* < 0.05. *** *p* < 0.001.

Although human pulmonary fibrosis entities are characterized and clearly distinguished by their histological patterns [47], there are certain fibrotic hallmarks found throughout these clinical entities [2,5,48]. Continuous deposition of ECM proteins by activated myofibroblasts leading to alveolar wall thickening [48] and replacement of alveolar tissue by connective tissue, mainly consisting of collagens [5,49] could be considered universal fibrotic motifs of the lung and were consequently found in IPF and AFE but not in control samples (Figure S2). These motifs, were analyzed in the PLK2 model. In general, the lung tissue of PLK2 KO mice appeared to be more dense but confluent, widespread fibrosis areas as observed in human IPF or AFE [47] were absent (Figure S2, Figure 3a,b).In PLK2 KO a significant increase of diffuse pulmonary collagen was found (Figure 3a,b,d,f,h). Compared to WT, the alveolar walls of PLK2 KO mice were significantly thicker and exhibited an irregular morphology (Figure 3c,f). Consequently, collagen deposition in the alveolar walls was found to be significantly increased in PLK2 KO compared to their WT littermates (Figure 3d,h). The prior in vitro characterization of pulmonary PLK2 WT and KO fibroblasts hinted at increased myofibroblast differentiation (Figure 1). Accordingly, immunohistochemical staining for αSMA revealed myofibroblast foci in PLK2 KO lung tissue (Figure 3e,i).

Taken together, PLK2 KO induces a spontaneous pro-fibrotic phenotype reflected by myofibroblast differentiation, fibrotic cytokine expression and certain histological alterations like alveolar wall thickening and increased collagen deposition. Published findings suggest that even subclinical fibrotic alterations of the pulmonary histoarchitecture should be monitored in patients as they may represent early stages of pulmonary fibrosis with the risk of disease progression and functional deterioration [50]. In this context, the PLK2 KO model could be used to better understand these early stage histological alterations. Furthermore, “multiple-hit models” have been proposed in lung fibrosis [51,52]. Although, genetic deletion of *PLK2* did not induce massive fibrosis as present in human, we found a clear pro-fibrotic substrate in which the effects of a “second hit” by, e.g., bleomycin [3,51] could be studied to identify fibrotic mechanisms and potential drug targets.

### 3.5. Pharmacological Inhibition of PLK2 Induces a Fibrotic Phenotype in Human Pulmonary Fibroblasts

**Figure 4:**
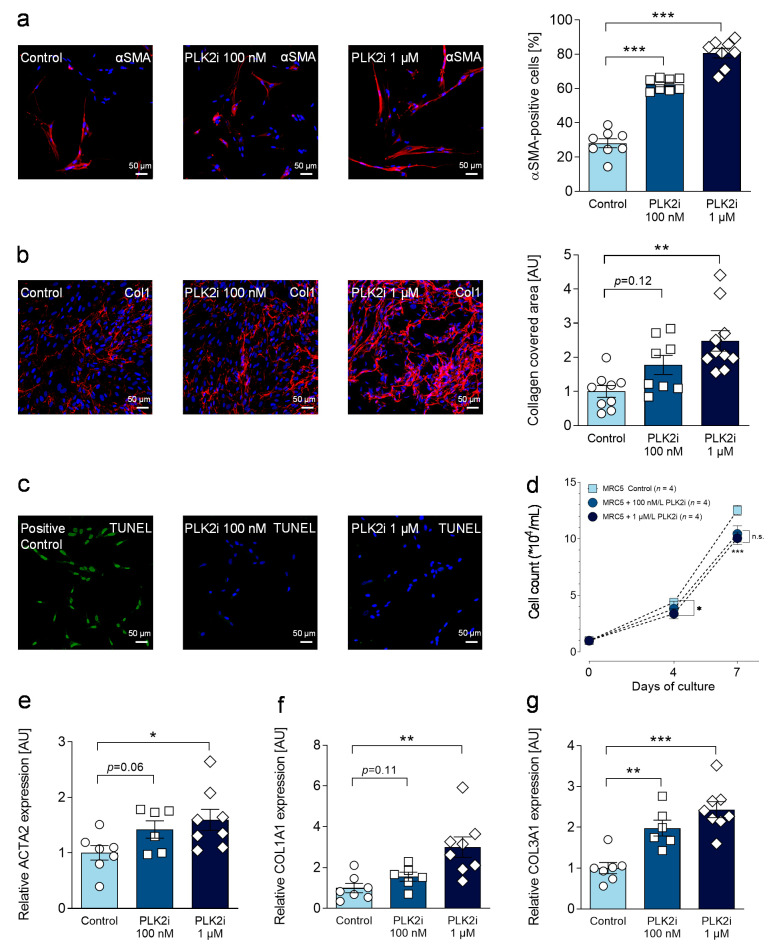
Effects of pharmacological PLK2 inhibition in human pulmonary MRC5 fibroblasts. (**a**) Quantification and representative immunofluorescence images of αSMA myofilaments in MRC5 fibroblasts under control conditions and in the presence of 100 nM or 1 µM specific PLK2 inhibitor (TC-S 7005) (*n* = 8 per group). The nuclei were stained with DAPI (blue). The scale bars equal 50 µm. (**b**) Quantification and representative immunofluorescence images of extracellular collagen 1 (Col1) deposition in MRC5 fibroblasts under control conditions and in the presence of 100 nM or 1 µM specific PLK2 inhibitor (TC-S 7005) (8 ≤ *n* ≤ 10). (**c**) Representative images of TUNEL staining to detect apoptosis. (**d**) Proliferation curves of MRC5 fibroblasts under control conditions and in the presence of 100 nM or 1 µM specific PLK2 inhibitor (TC-S 7005) (*n* = 4 per group). Cells were counted after 3 and 6 days. (**e**–**g**) Quantification of *ACTA2, COL1A1* and *COL3A1* gene expression determined by RT qPCR in MRC5 fibroblasts under control conditions and in the presence of 100 nM or 1 µM specific PLK2 inhibitor (TC-S 7005) (6 ≤ *n* ≤ 8). * *p* < 0.05. ** *p* < 0.01. *** *p* < 0.001. n.s. = not significant.

To examine, whether loss of PLK2 function induces direct pro-fibrotic changes in fibroblasts, we studied the effects of pharmacological PLK2 inhibition in a human pulmonary fibroblast cell line (MRC5). Fibroblasts were cultured for 72 h (24 h resting period after seeding and 48 h culture in the presence of solvent control, 100 nM or 1 µM PLK2 inhibitor) and one week (for proliferation experiments). Myofibroblast differentiation was significantly increased in a concentration-dependent manner (Figure 4a). Thus, secretion and deposition of extracellular collagen were similarly higher after 48 h of PLK2 inhibition (Figure 4b). Since we found significantly reduced proliferation rates in primary PLK2 KO fibroblasts, we sought to determine whether loss of PLK2 function induces apoptosis and thereby leads to reduced cell count. For this reason, a TUNEL assay was performed. Inhibition of PLK2 did not induce apoptosis (quantification displayed in Figure S4) in the tested concentrations (Figure 4c) but proliferation rates were significantly reduced after 3 days of culture at 1 µM and after 6 days at both concentrations (Figure 4d). These results are in line with previous studies claiming a critical role of PLK2 in cell cycle progression [53,54]. Lastly, gene expression of *αSMA*, *collagen 1* and *collagen 3* upon PLK2 inhibition was examined by RT qPCR and found to be closely aligned with the results of the functional experiments (Figure 4e–g). Taken together, pharmacological inhibition of PLK2 induced a pro-fibrotic phenotype conversion in human pulmonary fibroblasts thereby supporting the results obtained in primary PLK2 WT and KO fibroblasts.

### 3.6. Study Limitations

The present study has potential limitations, which have to be addressed in future research on this topic. The presented data suggests a role of PLK2 in human pulmonary fibrosis. Whether the observed downregulation of human *PLK2* expression is causal for or a consequence of pulmonary fibrosis development cannot be determined at this point. For human lung specimens, the sample size was relatively small because the availability of these specimens is limited due to medical and ethical reasons. A first characterization of the pulmonary phenotype of PLK2 deficient mice leads to the conclusion that lack of PLK2 function induces a pro-fibrotic cascade. Thus, restoration of PLK2 function and resulting prevention of fibrosis would be definitive proof of the hypothesis. Lastly, the interplay of molecular and cellular mediators, i.e., fibroblasts, immune cells and alveolar epithelium, as well as the mutual influence of the heart and the lungs need to be elucidated in future research to clarify which phenomena are causal and which are byproducts.

## 4. Conclusions

The present study identified a potential role of PLK2 in the pathophysiology of human pulmonary fibrosis. With a genetic predisposition towards pulmonary fibrosis and early stage pro-fibrotic alterations [50] of the alveolar histoarchitecture, the PLK2 KO model could be used in further research on this topic. Besides experimental fibrosis induction in vivo with, e.g., bleomycin or irradiation [3] in a “multiple-hit” model [51,52], ex vivo culture of PLK2 lung tissue and subsequent pharmacological interventions [55] could be feasible approaches towards the development of cost-and time-effective pulmonary fibrosis models and anti-fibrotic drug screening platforms.

## Figures and Tables

**Table 1:** Patient demographics.

	Control	IPF	AFE	OP	SSC
Male	3	3	3	3	3
Female	2	2	2	1	1
Age [y]	54.8 ± 7.8	53.4 ± 5.2	58.2 ± 1.9	58.25 ± 8.4	45.8 ± 1.9

IPF: idiopathic pulmonary fibrosis, AFE: alveolar fibroelastosis, OP: organizing pneumonia, SSC: systemic sclerosis.

**Table 2:** Primary and secondary antibodies.

Primary Antibodies				
Protein	Dilution	Conjugate/Source	Product-Nr.	Usage
αSMA	1:200	Mouse	A5228	ICC ^1^/ IHC ^2^
SMAD2/3	1:1000	Rabbit	#3102	WB ^3^
Phospho-SMAD2/3	1:1000	Rabbit	#8828	WB
ERK 1/2	1:1000	Rabbit	#9102S	WB
Phospho-ERK 1/2	1:1000	Rabbit	#9101S	WB
Ras	1:1000	Rabbit	#3339S	WB
Collagen 1 A1	1:100	Goat	MBS316282	ICC
Vimentin	1:200	Rabbit	ab137321	ICC
Discoidin Domaine Receptor 2 (DDR2)	1:200	Mouse	ab63337	ICC
**Secondary Antibodies**				
Goat-anti-rabbit	1:10,000	Peroxidase	111-035-045	WB
Alexa fluor 546	1:400	Streptavidin	Z25004	ICC
(Goat-anti-mouse)				
Alexa fluor 546	1:400	Streptavidin	Z25304	ICC
(Goat-anti-rabbit)				

^1^ Immunocytochemistry; ^2^ immunohistochemistry, ^3^ Western blot.

**Table 3:** Fibroblast immunofluorescence staining protocol.

Step	Action	Additional Information
1	Permeabilization (15 min, 0.1% Triton-X)	Room temperature
2	Washing (wash twice with PBS)	Room temperature
3	Blocking (1 h, 10% FCS)	Room temperature
4	Primary antibody (1 h in humidified chamber)	Room temperature or 4 °C overnight
5	Washing (wash twice with PBS)	Room temperature
6	Secondary antibody and DAPI (1 h in humidified chamber)	Room temperature
7	Washing (wash twice with PBS)	Room temperature
8	Mounting with 10 µL Fluoromount G	Room temperature
9	Storage until imaging	4 °C

**Table 4:** *PLK2* gene expression in different human pulmonary fibrosis entities.

	Control	IPF	AFE	OP	SSC
*n*	3	5	4	4	4
Mean	1.00	0.2	0.34	0.18	0.17
SEM	0.40	0.10	0.11	0.12	0.05
*p*-Value (vs. Control)	n.a.	0.020	0.038	0.023	0.021

n.a.: not applicable.

## Data Availability

All data generated in this study are depicted as individual values within the figures. Original data are available on reasonable request from the corresponding authors.

## Supplementary Materials

The following are available online at https://www.mdpi.com/2073-4409/10/3/617/s1. Figure S1: Detection of fibroblast marker proteins in PLK2 WT and KO primary fibroblasts; Figure S2: Pro-fibrotic gene expression and histological analysis of human Control, IPF and AFE lung sections; Figure S3: Full length blot for αSMA in primary PLK2 WT and KO fibroblasts; Figure S4: Quantification and original images of TUNEL assay for apoptosis detection.
