# Supplementary material for: Genetic Deletion of Polo-Like Kinase 2 Induces a Pro-Fibrotic Pulmonary Phenotype
Source: Cells. 2021 Mar 11;10(3):617. doi: 10.3390/cells10030617 (PMC8001503; doi:10.3390/cells10030617)
Supplement: Supplementary file 1 [file cells-10-00617-s001.pdf]

Supplementary Materials

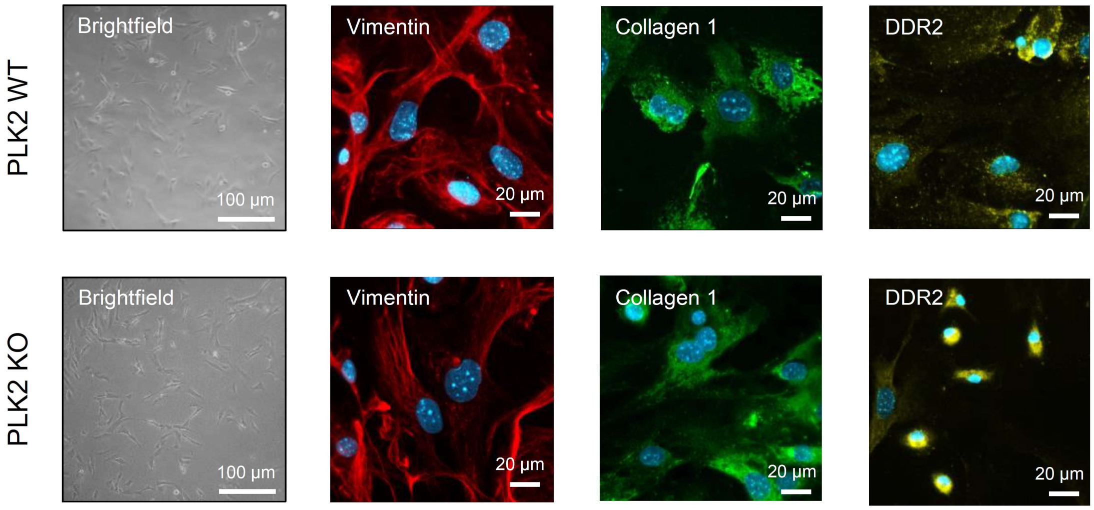

Figure S1. Detection of fibroblast marker proteins in PLK2 WT and KO primary fibroblasts. Morphological and immunocytochemical fibroblast identification. Representative brightfield and immunofluorescence images of the fibroblast markers vimentin, collagen 1 and DDR2 (pseudocolors were used for better discriminability). The nuclei were stained with DAPI (blue) and the scale bars equal 100 and 20 µm, respectively.

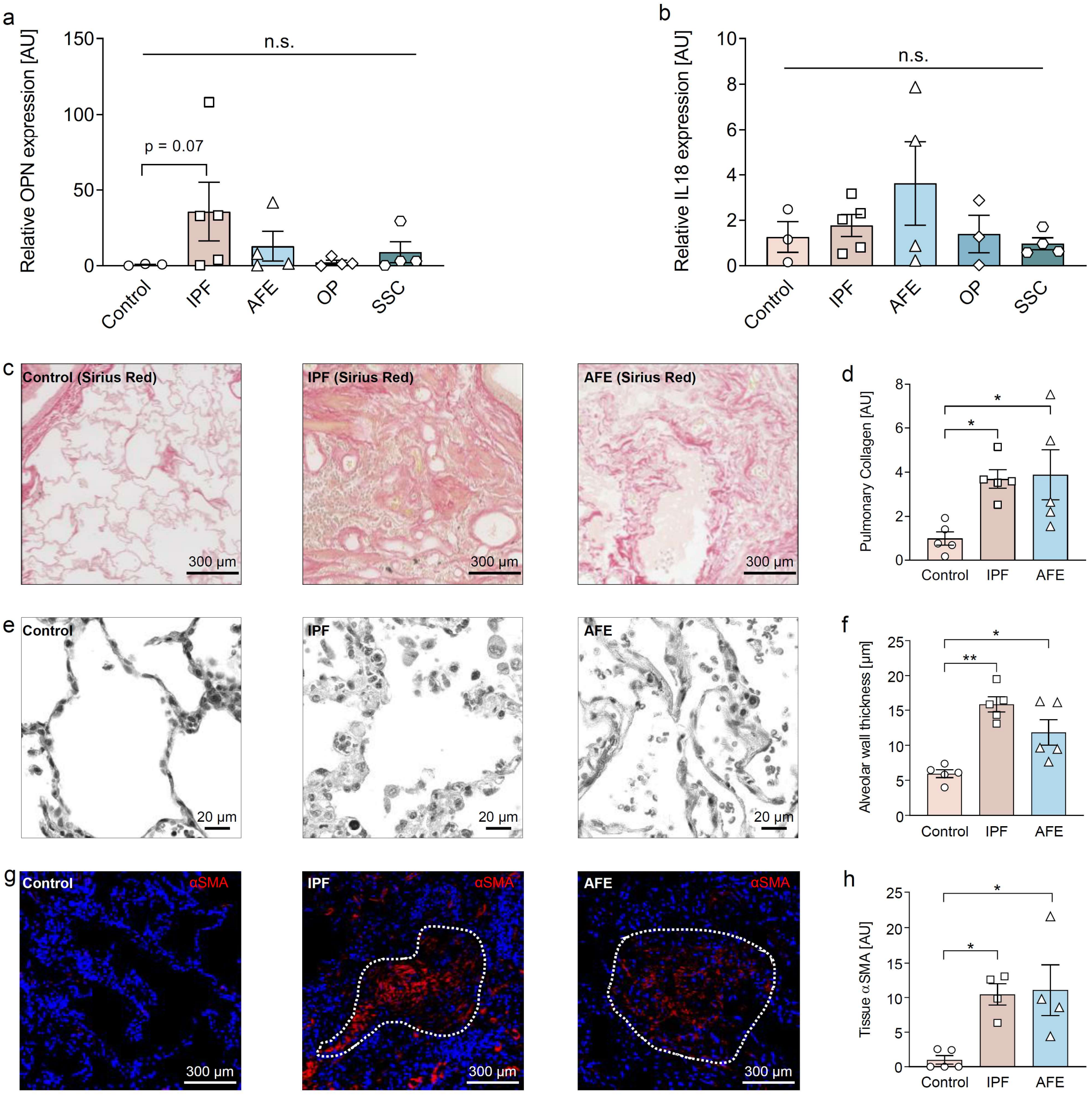

Figure S2. Pro-fibrotic gene expression and histological analysis of human Control, IPF and AFE lung sections. (**a**) Quantification of OPN gene expression (determined by RT qPCR) in human lung tissue normalized to Control ($n_{control}$ = 3, n_{IPF} = 5, n_{AFE} = 4, n_{OP} = 4, n_{SSC} = 4). (**b**) Quantification of IL18 gene expression in human lung tissue normalized to Control ($n_{control}$ = 3, n_{IPF} = 5, n_{AFE} = 4, n_{OP} = 3, n_{SSC} = 4). (**c**) Representative images of picrosirius red collagen staining in human lung sections. The scale bars equal 300 µm. (**d**) Fibrosis quantification ($n_{control}$ = 5, n_{IPF} = 5, n_{AFE} = 5). (e) Representative images of human alveolar tissue (black and white for better contrast visualization). The scale bars equal 20 µm. (**f**) Quantification of alveolar wall thickness [µm] ($n_{control}$ = 5, n_{IPF} = 5, n_{AFE} = 5). (**g**) Representative immunofluorescence images of αSMA (red) and nuclei (blue) in human lung sections. Highlighted areas (dashed white outline) show myofibroblast accumulation (foci). The scale bars equal 300 µm. (**h**) Quantification of αSMA fluorescence intensity (normalized to nuclei) in in human lung sections ($n_{control}$ = 5, n_{IPF} = 4, n_{AFE} = 4). * $p < 0.05$. ** $p < 0.01$.

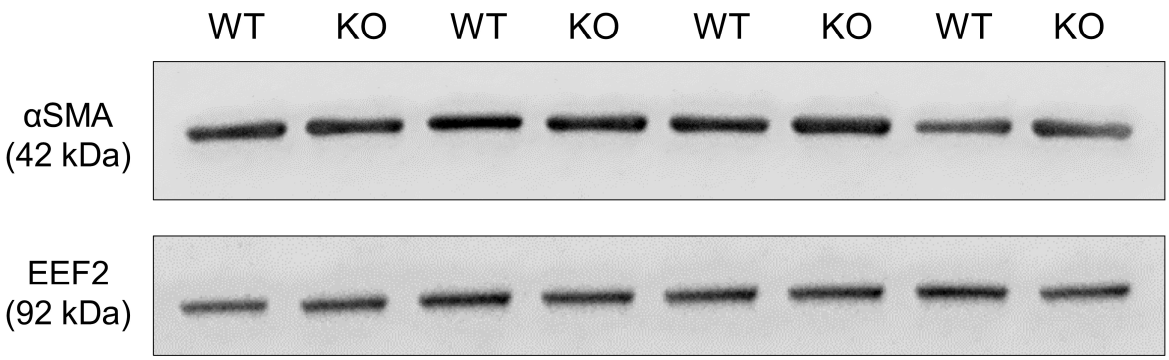

Figure S3. Full length blot for αSMA in primary PLK2 WT and KO fibroblasts.

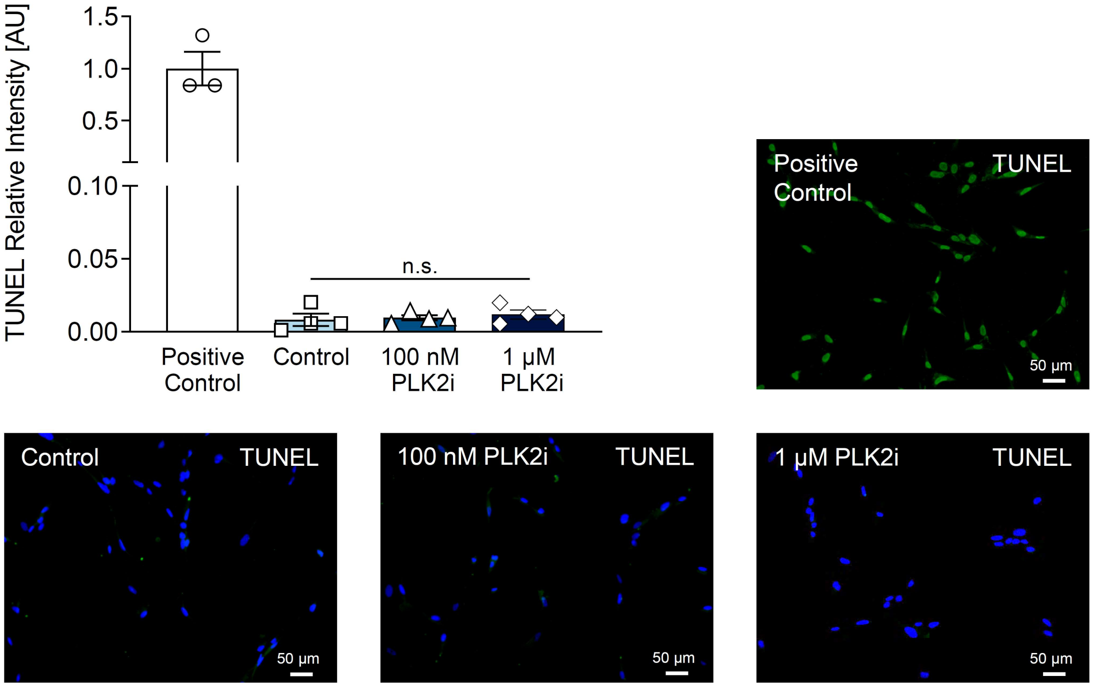

Figure S4. Quantification and original images of TUNEL assay for apoptosis detection.
